# The experiential perspectives of siblings and partners caring for a loved one with an eating disorder in the UK

**DOI:** 10.1192/bjo.2022.43

**Published:** 2022-03-24

**Authors:** Rachel Batchelor, Hannah Cribben, Pamela Macdonald, Janet Treasure, Erica Cini, Dasha Nicholls, Carol Kan

**Affiliations:** Department of Psychology, Royal Holloway, University of London, UK; Institute of Psychiatry, Psychology & Neuroscience, King's College London, UK; Institute of Psychiatry, Psychology & Neuroscience, King's College London, UK; Institute of Psychiatry, Psychology & Neuroscience, King's College London, UK; East London Eating Disorder Service for Children and Young People, East London NHS Foundation Trust, UK; and Nutrition Science Group, Division of Medicine, University College London, UK; Division of Psychiatry, Imperial College London, UK; Institute of Psychiatry, Psychology & Neuroscience, King's College London, UK

**Keywords:** Eating disorders, carers, siblings, partners, service provision

## Abstract

**Background:**

Caring for a loved one with an eating disorder typically comes with a multitude of challenges, yet siblings and partners are often overlooked. It is important to understand if current clinical guidance for supporting carers are effective and being utilised for these groups, to help meet their needs.

**Aims:**

To identify the experiential perspectives of siblings and partners of a loved one with an eating disorder compared with guidance for improving the adequacy of support provided to carers published by Beat and Academy for Eating Disorders.

**Method:**

Three online focus groups were held for ten siblings and five partners from across the UK (12 females and three males). Carers had experience of caring for a loved one with anorexia nervosa (13 carers) or bulimia nervosa (two carers), across a range of therapeutic settings. Focus group transcriptions were analysed with thematic analysis.

**Results:**

Four key themes were identified: (a) role-specific needs, (b) challenges encountered by siblings and partners, (c) generic needs and helpful strategies or approaches, and (d) accounts of service provision and family support.

**Conclusions:**

Overall, the majority of experiences reported by siblings and partners did not meet the published guidance. Consequently, clinical practice recommendations were identified for services, alongside the charity sector, to take a proactive approach in detecting difficulties, providing skills training and emotional/practical support, adapting/tailoring peer support groups and supporting online facilitation. Our findings part-informed the design of our national online survey on loved ones’ experiences of care in eating disorders.

Eating disorders are among the most common serious mental health conditions, with an estimated prevalence of 1.25 million in the UK.^1^ Providing support for a loved one with an eating disorder can play a crucial role in improving the well-being, recovery and prognosis of patients with eating disorders.^2^ Indeed, carers can encourage help-seeking behaviours, foster recovery motivation, contribute to positive self-concept development and strengthen self-esteem, all factors that have been shown to improve therapeutic outcomes.^3,4^ Moreover, loved ones can advocate for individuals with eating disorders, ensuring they receive a high quality of care with access to evidence-based treatments.^3^ Despite such positives, the challenge and responsibility of caregiving can make carers vulnerable to poorer physical and mental health, as well as feelings of guilt, loneliness, loss, uncertainty and worry.^5,6^ These negative effects on carers can then adversely affect the treatment outcomes of people with eating disorders, highlighting the need for providing carers with appropriate support.^6^ The National Institute for Health and Care Excellence guidelines for eating disorders^7^ specifies that family members who perform the role of caregivers, and therefore require support, include the siblings and partners of people with an eating disorder. Therefore, all close relationships, such as parent–child, sibling and intimate adult relationships, are typically affected and reorganised in the presence of an eating disorder, with the illness typically taking a central position.^8^ However, previous literature exploring the needs and perspectives of carers has primarily focused on parents.^5^ Thus, other common groups of carers, including siblings and partners, have remained overlooked, despite recent research showing that the adverse effects of being a carer for a loved one with a serious mental health condition are not limited to parents.^9–12^ To improve the adequacy of support provided to carers, both Beat (the leading UK eating disorder charity)^13^ and the Academy for Eating Disorders (AED; the leading association for eating disorder professionals and experts with lived experience)^14^ recently published guidance outlining recommendations on improving the adequacy of support provided to carers such as siblings and partners. (Table 1). The guidelines include recommendations for carer involvement, communication with services, information, resources and support, the assessment of carers’ needs and monitoring of their well-being, peer support, skills training, transition support and feedback processes.^13,14^ However, the extent to which these guidelines are being adhered to has not yet been investigated. Such research is important, as identifying what changes still need to be implemented has potential to improve the experiences of siblings and partners, as well as outcomes for both carers and patients with eating disorders. Therefore, this study aims to explore the experiential perspectives of siblings and partners of a loved one with an eating disorder, and compare this with the best-practice standards and healthcare rights published by Beat and AED, respectively. Combined with information gathered from our paper on the experiential perspectives of parents,^15^ this information will inform the design of a large-scale national online survey to explore the experiences and needs of those caring for a loved one with an eating disorder across the UK.

**Table 1:** Published guidelines for meeting the needs of families and carers affected by eating disorders

Academy for Eating Disorders
Access to Quality Care: All patients have the right to immediate care for medical and/or psychiatric instability, followed by timely and non-discriminatory access to appropriate specialty care.
Respect: All patients, caregivers and family members have the right to be treated with respect throughout the assessment, planning and treatment process. Patients and carers should never be judged or stigmatised based on symptoms, behaviours or past treatment history.
Informed consent: When making healthcare decisions, patients and caregivers have the right to full disclosure by healthcare professionals about treatment best practices, risks, costs, expected service outcomes, other treatment options and the training and expertise of their clinicians.
Participation: Families and other designated carers have a right to participate in treatment as advocates for the best interests of their loved ones. Caregiving responsibilities and degrees of participation will necessarily vary depending on the age, mental state and diagnosis of the patient, as well as the caregiver's skills, availability, personal health, resources and other circumstances.
Communication: All patients and carers have the right to establish regular and ongoing communications through clearly defined channels. Caregivers and family members have the right to communicate their observations and concerns to professionals, and to receive information when the patient's medical stability and/or psychiatric safety is threatened or at risk.
Privacy: All patients and carers have a right to expect their health professionals to understand, communicate and respect the applicable privacy or age-of-consent regulations that govern the communication of health and treatment information, as well as the circumstances and conditions that may override privacy concerns or transfer authority regarding treatment decisions.
Support: All caregivers have a right to receive information, resources and support services to help them understand and carry out the expectations and responsibilities of their roles as partners in treatment.
Beat
Have a policy that ensures optimum involvement of, and support for, all carers as soon as a loved one starts treatment.
Train all service staff in the application of the policy and these standards, with particular focus on the importance of carers as a resource for recovery.
Provide all carers with useful and comprehensive information about eating disorders when their loved one receives a diagnosis.
Offer all carers and siblings an assessment of their own needs when a loved one receives an eating disorder diagnosis, continue to monitor their well-being throughout the their treatment and, where necessary, refer carers to specialist services.
Offer all carers options for peer-to-peer support.
Offer all carers opportunities to learn the necessary skills to provide optimum support for their loved ones.
Inform and engage carers when a loved one faces a transition between services, and ensure that effective communication between both services and carers takes place.
Provide a mechanism by which carers’ input and feedback is sought and acted upon.

## Method

### Participants and sampling

Siblings and partners of individuals with an eating disorder were recruited via local and national eating disorder advertisements. Inclusion criteria were that participants should be aged ≥18 years and the partner or sibling of a loved one who had received treatment for an eating disorder in the UK in the past 10 years. Following recruitment, no participants dropped out of the study.

In total, ten siblings (nine females and one male) and five partners (two females and three males) participated. Focus groups 1 and 2 consisted of five siblings each, and a third focus group consisted of the five partners. All participants were of White ethnicity, and the majority (*n* = 11; 73%) were aged between 21 and 30 years at the time of focus group participation. All participants lived in either England (*n* = 12; 80%) or Scotland (*n* = 3; 20%). The demographic and illness-related characteristics of their loved one with an eating disorder are displayed in Table 2.

**Table 2:** Demographic and illness-related information of participants’ loved ones with an eating disorder

Characteristic	Loved one with an eating disorder (*N* = 15)
Gender, *n* (%)	
Male	3 (20.0)
Female	12 (80.0)
Age, mean (s.d.; range)	26.93 (11.49; 14–61)
Eating disorder diagnosis, *n* (%)	
Anorexia nervosa	13 (86.7)
Bulimia nervosa	2 (13.3)
Illness duration in years, mean (s.d.; range)	8.13 (9.80; 0.5–40)
Treatment type accessed, *n* (%)	
In-patient	9 (60.0)
Day care	4 (26.7)
Out-patient	12 (80.0)
Treatment age-band accessed, *n* (%)	
Child and Adolescent Mental Health	7 (46.7)
Adult	2 (13.3)
Both	6 (40.0)

### Procedure

This study was performed in accordance with the Declaration of Helsinki. This study was approved by King's College London (approval number HR-19/20-14803). All participants provided written informed consent to participate. Because of COVID-19 restrictions, the three focus groups were hosted online via Microsoft Teams for Mac (Version 16.35). The focus groups were semi-structured; participants were initially asked if they had previously seen the guidelines and then discussed their perspectives and experiences of the recommendations that they felt were most important. The topic guide (Appendix 1) and additional details of the methodology used for these focus groups are described elsewhere.^15^ Participants received a £20 voucher for their time.

In line with participatory research methodologies,^16^ a patient and public involvement group (made up of professionals and carers) were involved in all stages of the research, including designing the topic guide for the focus groups in this study. Moreover, an individual with lived experience of caring for a sibling with an eating disorder was viewed as an expert by experience, and was involved in all stages of this research. This included data analysis and manuscript writing, to improve representation and sensitivity. We felt a participatory approach was especially important for this project, as siblings and partners are commonly overlooked caring groups. This has led to a paucity of research in this area. Their involvement in all stages of the study helped to ensure our project remained important and relevant to siblings and partners.

### Analysis

An inductive approach to thematic analysis was used at the semantic level.^17^ To address potential researcher bias, an independent researcher (P.M.), who was not involved in the study design or focus group participation, coded the transcripts alongside two other researchers within the team (R.B. and H.C.). Utilising multiple researchers, with a mix of clinical and lived experience expertise, to independently code and analyse our rich data led to the unanimous results and increased the reflexivity and rigour of the study.

In the initial analysis phase, three researchers (R.B., H.C. and P.M.) familiarised themselves with the data through re-reading the transcripts and noting interesting aspects. The three researchers identified potential codes for each focus group independently, and then met to discuss inconsistencies. Initial themes and subthemes reflecting broad units of common ideas were formed by grouping relevant codes together. In the final phase, the researchers consolidated and clearly defined the themes and subthemes, and their interrelated links, over several meetings.

Iterative analysis of the transcript showed that saturation of data was reached, as the final focus group transcript produced no new themes or subthemes. The final thematic framework was then devised over a series of four meetings between the research team. Analysis was carried out using NVivo for Mac (Version 12, QSR International, Doncaster, Australia; see https://www.qsrinternational.com/nvivo-qualitative-data-analysis-software/support-services/nvivo-downloads).

## Results

**Fig. 1:**
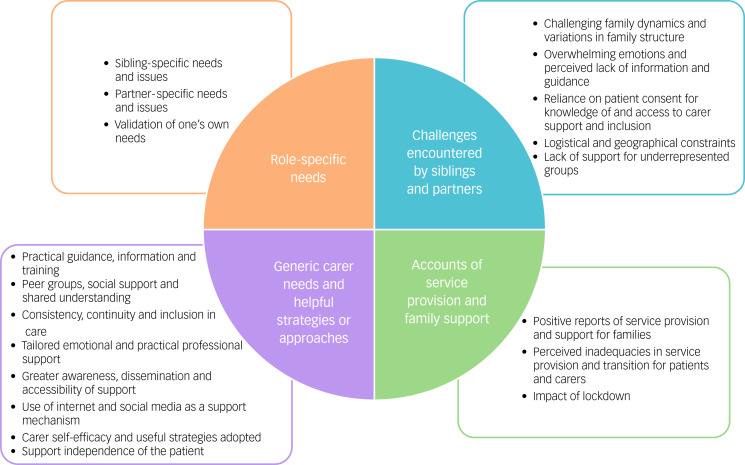
Overview of themes and subthemes identified from focus groups, using thematic analysis.

Four main themes from the three data-sets were identified and are outlined alongside the subthemes in Fig. 1.

### Role-specific needs

This theme covers needs unique to specific family members, namely partners and siblings.

Sibling-specific needs and issues were described by siblings in our focus groups to address the gap in the support system that helps siblings. Participants expressed that having resources specifically designed for siblings (e.g. on topics such as such as managing sibling relationships in the context of an eating disorder and taking on a caring role as a sibling) would be more helpful than the generic guidance for carers that they had seen, which was often geared toward parents. Suggestions included support, guidance and practical training tailored toward siblings.

‘I think having more stuff that's specifically around siblings and what a sibling's role might be would be helpful.’ Focus group 2, sibling, female.

‘I kind of fitted into the parents when they were having discussions, um which I struggle with because I don't feel I have the, not authority, but parents have more a say for my sister than I do. So, the information they gave my parents is different for me and I don't feel like I got the support in that way.’ Focus group 2, sibling, female.

‘I feel like quite often carers is just read as parents and siblings are still overlooked so if the word siblings could at least be in there somewhere, such as parents and siblings, ideally something specific for siblings.’ Focus group 1, sibling, female.

Partner-specific needs and issues were highlighted by participants, including issues with navigating life with an eating disorder within an intimate adult-to-adult relationship, and difficulties surrounding choice of whether to remain with their partner throughout periods where they had seemingly changed as a result of being so unwell. A need for couple's therapy was also highlighted, as well as resources specifically tailored toward partners.

‘ … So you have a carer-patient relationship which is not dissimilar to a parent-child relationship, it can be very difficult to get back to an adult-adult relationship from there.’ Focus group 3, partner, male.

‘ … We need to also look after our relationship and relationships are just entirely forgotten in this process and I appreciate that is probably beyond the scope of what you do but getting that relationship part right post-treatment will aid with recovery and help with the relapse because you know.’ Focus group 3, partner, male.

‘ … Having that clinical support of just really reinvigorating your toolkit as a partner and how you help is very useful, I think.’ Focus group 3, partner, female.

Participants reflected upon their desire for validation of one's own needs and the importance of looking after one's own psychological well-being. Further narratives highlighted the need of services taking a proactive approach themselves to reaching out to siblings and partners.

‘ … It can be quite hard to kind of feel like your problems are valid enough to seek support yourself or even just knowing that someone is out there, sort of monitoring you whilst you're supporting someone else can be quite validating, I think.’ Focus group 1, sibling, female.

‘ … Having someone actually say, “are you OK?” I think it would make a big difference.’ Focus group 1, sibling, male.

### Challenges encountered by siblings and partners

There were several subthemes representing the challenges encountered by carers. Siblings reflected upon addressing challenging family dynamics and variations in family structure, including divided loyalties, trust issues, familial stress because of the eating disorder and feelings of exclusion by certain family members because of focus on the ill person.

‘ … My sibling shares a lot more with me than she does with our parents but that can be quite a huge burden because it's like “aww man how do I not compromise her trust, but also if I'm told something concerning how, what do I do with that information?”’ Focus group 1, sibling, female.

‘ … The stress that everyone is feeling makes the relationships in the family really difficult as well, when there's one person who is so ill in the family.’ Focus group 2, sibling, female.

There were also some positive accounts. For example, some participants described how different family members found their own way to support each other.

‘We [my well sister and I] both managed to support each other really really well in that way because we both could see different sides of things, we've always been quite different but we do, I think together she can say something and I can think oh I've not actually noticed that and then we sort of work together to be able to help each other and rationalise each other.’ Focus group 2, sibling, female.

‘I've got three sisters … and for us we did have peer support from each other because, you know, the three of us were trying to deal with it, but if we hadn't had that it would have been really difficult.’ Focus group 2, sibling, female.

Participants described overwhelming emotions and perceived lack of information and guidance, such as strategies for providing adequate support. Siblings also noted difficulties with applying the information they had read online to their individual circumstances. Overwhelming emotions described by siblings included stigma and guilt over feeling that they were not providing their loved one with adequate support and that they were struggling and in need of some support for themselves.

‘At the start you're sort of thrown into it you don't have any hand holds or foot holds or paths with directions of which way to go you just have to kind of figure it out.’ Focus group 1, sibling, male.

‘ … I felt so guilty for reaching out for help because I wasn't the one who was struggling here, it was my sibling.’ Focus group 1, sibling, female.

Partners described overwhelming feelings of helplessness, uselessness and self-blame over not doing more to support their partner and help them to access the services they needed to. Feelings of loneliness were also expressed. Furthermore, some partners described accounts of living with problematic behaviour and thoughts, such as loved ones not acknowledging that there was a problem.

‘ … You can feel so helpless as a partner.’ Focus group 3, partner, female.

‘I think when she relapsed in terms of blaming myself in that I wasn't able to support my partner enough that she was going to slip through the net and I should have been there for her enough.’ Focus group 3, partner, male.

‘Or they are denial, because my wife wouldn't acknowledge there was a problem, everybody else could see it but she couldn't.’ Focus group 3, partner, male.

Narratives referred to the reliance on patient consent for knowledge of and access to carer support and inclusion to any carer groups, such as family therapy. Both confidentiality issues regarding sharing patient information with carers in adult services and patient reluctance to either engage in, say, family therapy or interventions that include involving the family were referred to in this subtheme.

‘When my sibling was an in-patient, that information came through her, so it was dependant on her telling us that there were these things available.’ Focus group 2, sibling, female.

‘ … They had my number and I had their number sort of thing which she had agreed to confidentiality so I was allowed to know sort of what was going on, but … at the time they might not want you to be involved or sort of want you know what's happening and that completely makes sense there could be some problems with confidentiality as well.’ Focus group 1, sibling, female.

‘[My sibling] was really resistant to that, like I said before she didn't particularly like having the therapy anyway so having us involved, she was quite resistant to that.’ Focus group 1, sibling, female.

Participants outlined the logistical and geographical constraints encountered when supporting a loved one with an eating disorder; for example, accessibility of support groups when at university and geographical challenges.

‘I'd come back on uni holidays and things and that was really hard, um because you weren't living with it every day you just had to deal with it when you came back.’ Focus group 1, sibling, female.

Reports suggested there was a lack of support for underrepresented groups, such as siblings and partners caring for older people with eating disorders, complex and enduring cases or male patients with an eating disorder.

‘There is not a lot of material out there as a man with an eating disorder.’ Focus group 3, partner, female.

‘ … Particularly for older or complex patients the scope of care is insufficient.’ Focus group 3, partner, male.

### Generic carer needs and helpful strategies or approaches

This theme incorporates needs that are applicable to all people caring for a loved one with an eating disorder and perceived strategies, tools and approaches deemed helpful to the caring role. These needs included practical guidance, information and training on how best to support their loved one. Suggestions included psychoeducational guidance, support, resources, training videos and general training on how to respond to the various scenarios that commonly occur when supporting a loved one with an eating disorder.

‘ … Video resources where you can access someone sort of explaining it to you, because I think it's much more easily explained than it is read.’ Focus group 1, sibling, male.

‘The most important one for me would be to provide carers with more comprehensive information about the eating disorder.’ Focus group 3, partner, male.

‘I'd find a pack quite frankly a bit condescending and trite.’ Focus group 3, partner, male.

Participants indicated the helpfulness of peer groups, social support and shared understanding. This included peer support groups, in either a face-to-face or online group format, as well as perceived helpful social support from family, friends and peers. Some participants reflected on their previous use of peer support, particularly through social media, whereas others perceived it as a potentially helpful and comforting avenue of support. There were also several examples of empathy and support provided to each other within the study focus groups. Some participants shared an appreciation for being able to talk and listen to others outside of their family network who understood some of what they were going through. A preference for professional moderation with usage guidelines for support groups was also noted, as well as having some consistency in the attendees to enable familiarity and connections to form. Some narratives referred to negative aspects of support groups, such as becoming worried when hearing the experiences of others or being of a different demographic (e.g. a lot older) than others within a peer support group and thus finding it difficult to relate and connect. There was a link between this subtheme and ‘use of the internet and social media as a support mechanism’, since many support groups are online based.

‘In all honesty it's been amazing coming to be here and listen to everybody else's different experiences and stuff so thank you because it gives me hope.’ Focus group 3, partner, female.

‘ … They were all just parents and I think I could feel a generational gap and it would be so much nicer to be able to get support from people my age.’ Focus group 1, sibling, female.

Many reports highlighted the need for consistency, continuity and inclusion in care. These ranged from having access to a team of professionals who have first-hand knowledge and experience of the treatment of the loved one to continuity throughout the process, such as from in-patient treatment through discharge, and the importance of feeling included in care.

‘I'm not sure how that would work in practice but just something more consistent where you can build up some kind of trust with the people you are talking to.’ Focus group 1, sibling, female.

‘ … It would have been invaluable for me to be able to feel more included in the process.’ Focus group 3, partner, male.

Participants recognised that everyone's experiences are different and therefore expressed a desire for tailored emotional and practical professional support, to address one's individual set of circumstances with regard to both the loved one with the eating disorder and their family, rather than generic support, which may not apply.

‘ … Being able to actually ask someone else questions is also really important because obviously everyone else's experiences will be so different.’ Focus group 2, sibling, female.

‘ … I think most of us would recognise that this is a very specific manifestation on a case-by-case basis and sometimes you really do need to be told what's the best personal thing you can do for your patient, not the population. So, I felt it needed a bit more specific contact throughout the treatment.’ Focus group 3, partner, female.

There was a suggestion for greater awareness, dissemination and accessibility of support for families and carers, along with detailed information on how to access such support, such as the Beat guidelines, being made more accessible.

‘I think just being made aware of what is available would be really useful.’ Focus group 2, sibling, female.

Narratives referred to the use of the internet and social media as a support mechanism. There was a link between this subtheme and the ‘peer groups, social support and shared understanding’ subtheme as several references to peer group support consist of online forums, such as those organised by Beat.

‘One major source of support for me was actually Instagram, it was following other siblings who had similar experiences to me … ’ Focus group 1, sibling, female.

There was evidence of carer self-efficacy and useful strategies adopted by carers to improve their own well-being and situation with their loved one. Examples included seeking independent support from external bodies, such as pastoral care at university, therapy from work or other support independent of the patient's family or care teams. Accounts also reflected upon what carers deemed important in the recovery process, such as open and honest communication for partners.

‘I did seek help, like counselling services whilst at university.’ Focus group 1, sibling, female.

‘I'm very outgoing in trying to educate myself on bulimia … ’ Focus group 3, partner, female.

Responses for siblings emphasised the importance of attaining support independent of the patient, because many of their issues and concerns were related to the impact that the eating disorder has had on the patient and the family. Accounts highlighted the need for siblings to be regarded as a resource in the recovery process, as well as recognition of their own need to be involved.

‘I think if the only option is to go through her, that wasn't something we could do.’ Focus group 1, sibling, female.

‘ … There were definitely things where if she was present, I wouldn't say because it might reflect badly on her, she might feel bad about how sometimes the family feel, it makes them sort of embarrassed to share, I think it can be quite traumatic for the family, so it's like I think it's really important that whatever the support is it's very separate.’ Focus group 1, sibling, female.

### Accounts of service provision and family support

This theme encompasses responses that represent any aspect of service provision. There were some positive reports of service provision and support for families regarding the availability of support being offered to the family within the service provision package, both during in-patient stay and the transition period. The importance of family inclusion was emphasised. Examples of helpful support included (multi-)family therapy, having a care coordinator, having a direct line of contact with the treatment team and workshops on how to support their loved one.

‘We did something called multi-family therapy which was through CAMHS [Child and Adolescent Mental Health Services], which was um 4 days, 5 days, really intense, but really helped.’ Focus group 2, sibling, female.

‘I felt that when my wife was in there was a lot of support. They made sure I understood what was going on all the way, it was a good experience.’ Focus group 3, partner, male.

Some participants reflected upon perceived inadequacies in service provision and transition for patients and carers received throughout the various stages of treatment, such as long waiting lists and difficulties obtaining referrals and initial diagnoses, and the adverse effects this had on the patients and their loved ones trying to support them. Lack of adequate support for both patients and carers during periods of transition between services, such as between in-patient/day patient care and out-patient care, was also highlighted.

‘We're on a waiting list for the third time and there's … we've been on this waiting list for over a year but each time we have to go through all the hoops again of the GP [general practitioner], the local mental health and quite frankly those channels are not fit for purpose for eating disorders.’ Focus group 3, partner, male.

‘ … They never gave me any information or advice giving or anything like that.’ Focus group 2, sibling, female.

Moreover, responses described the impact of lockdown and COVID-19 on the care that families received from services.

‘I've seen a big change during lockdown and obviously we've not been able to go to in-patient, it's always been like through videocall, and I think that's also made it difficult.’ Focus group 1, sibling, male.

## Discussion

This study sought to explore the experiential perspectives of siblings and partners caring for a loved one with an eating disorder compared with published clinical guidance. We identified four main themes summarising the perspectives of ten siblings and five partners of a loved one with an eating disorder: (a) role-specific needs, (b) challenges encountered by siblings and partners, (c) generic needs and helpful strategies or approaches, and (d) accounts of service provision and family support. Some positive experiences of service provision and support for families were shared by siblings and partners, in line with the guidance from Beat and AED.^13,14^ For example, some reflected on the good standards of care their loved one received, as well as some positive experiences of family support being included within the service provision package and a recognition from services of the importance of family inclusion. Other examples of helpful support for carers reported included access to a care coordinator, multi-family therapy and workshops for supporting their loved one.

The study also identified several challenges encountered by carers across a range of treatment settings and at various stages of treatment. Some of these challenges were in keeping with those commonly discussed within previous literature on eating disorder service provision, such as poor support for carers during transition periods (for example, from in-patient/day patient care to out-patient care) and a need for greater support for underrepresented eating disorder groups, namely males and older women, and their carers.^18–20^ This suggests the guidance is not being followed regarding engaging and effectively communicating with carers during transitions and providing non-discriminatory access to appropriate specialist care.^11,12^ Our findings also reflected challenges commonly identified by carers of loved ones with an eating disorder, including overwhelming emotions and lack of information and guidance.^21^ These findings were despite both the Beat and AED guidelines noting the need for carers to be provided with useful and comprehensive information, resources and support.

Notably, our findings built upon our current understanding of the challenges of caring for a loved one with an eating disorder that are particularly relevant to sibling and partners. For siblings, this included the management of sibling relationships in the context of an eating disorder, as well as effects on family dynamics and their position in the family, with feelings of responsibility and divided loyalties being identified.^9,21,22^ Moreover, some siblings described logistical and geographical constraints in supporting their sibling, such as moving away from the family home to university. For partners, difficulties navigating intimate adult-to-adult relationships with a loved one with an eating disorder, coping with the changes in the person they first fell in love with as a result of the eating disorder, as well as the element of choice surrounding remaining with their partner, were expressed.

Considering many siblings and partners felt ‘overlooked’, guilty for seeking help and reliant on loved ones for support and inclusion, our participants emphasised the need of a more proactive approach from services by reaching out to partners and siblings, validating their needs and offering appropriate support. Indeed, the best-practice standards by Beat^13^ stress the importance of offering all carers, including siblings, a needs assessment and continually monitoring their well-being to allow timely referral to appropriate specialist services as necessary. This may include offering social support and learning opportunities. Likewise, AED^14^ outlines the right for carers to have regular and ongoing communications with professionals, as well as to receive information, resources and support services to enable them to understand and carry out their caring responsibilities.

Collectively, despite some positive experiences, our findings highlight several unmet needs of siblings and partners of loved ones with an eating disorder when compared with published guidelines, indicating a need for closing these gaps in support. In light of eating disorder services being over-stretched, particularly since the COVID-19 pandemic, this could be an area for collaboration between eating disorder specialist healthcare providers and the charity section, so that together, the range of needs of all carers (parents, siblings and partners) are considered and met.

### Clinical implications

Our focus groups gathered the unique personal perspectives of siblings and partners and generated several recommendations for moving toward the best-practice standards and healthcare rights published by Beat and AED, respectively. Recommendations for fostering a more proactive approach included increasing the dissemination (e.g. advertisement and accessibility) of the support available and making healthcare professionals and services more mindful of carer groups beyond parents. Regularly screening for concerns among siblings and carers could also be used to flag difficulties to care teams. Such a proactive approach may also reduce the need for siblings and partners to seek their own alternative support, which may be less eating-disorder specific, from avenues such as work or university. This could help to ensure that emotional and practical professional support is specialist, as recommended by Beat,^13^ and tailored to the individual circumstances, something that was highlighted as important in our sample.

Our sample identified that practical guidance, information and training would be beneficial, in accordance with recommendations from Beat^13^ and AED.^14^ Previous research has highlighted the benefits of such an approach; the New Maudsley collaborative care intervention provides carers with theoretical and practical knowledge for coping with eating disorders.^23^ This approach has been successful both in the form of a workshop and self-help guides, providing choice and flexibility. Some skill-based self-help guides for carers^24,25^ also have sections specifically aimed at siblings and partners, providing tailored and accessible guidance.

Greater access to couple's therapy was also recommended for partners, to address the difficulties described by partners, namely navigating intimate adult-to-adult relationships with an eating disorder. Thus far, empirical literature has supported the treatment of adults in a couple's context, and an increasing evidence base has shown promising relational and therapeutic outcomes for couple's-based interventions for adults with eating disorders.^26,27^

In line with previous research, seeking social support was also identified as a way of coping.^21^ Previous research has shown the benefits of peer support groups for carers including siblings and partners, such as sharing experiential knowledge and mutual understanding.^12,28,29^ Building upon Beat's recommendation of offering carers options for peer support, our sample indicated support groups tailored for siblings or partners might be beneficial, given their experiences of attending groups that had been primarily populated by parents. A preference of group attendee consistency to build connections and professional moderation were noted. To reduce logistical and geographical constraints associated with accessing such support, our participants suggested online delivery methods and use of social media.

Further practical steps that healthcare providers, alongside the charity sector, could collaboratively undertake to meet the guidance outlined by Beat and AED include ensuring that all services have easily accessible resources specifically aimed at siblings and partners; having a named person who understands the sibling/partner's circumstances and can be contacted, irrespective of geographical distances, such as university; and involving siblings and partners in their loved one's journey, where appropriate.

### Limitations

Given the long duration of the eating disorder for the loved ones in most of our sample, it is likely that they had experience of healthcare services before 2017 and 2019, when the AED healthcare rights and Beat best-practice standards were respectively published, and indeed before the investment of Community Eating Disorder Services for Children and Young People (CEDS-CYP) in 2016. Therefore, it is difficult to determine the level of influence the guidelines and investment may have had. That said, it is noteworthy that most of our participants’ loved ones were still accessing services, and most participants had no previous awareness that these publications existed.

Furthermore, our analyses did not yield a direct comparison between services, making it difficult to ascertain whether our findings are equally relevant across settings. For example, as family therapy is more common in CEDS-CYP compared with adult mental health services,^30^ it is possible that CEDS-CYP facilitate sibling involvement to a greater extent. Additionally, 80% of partners had only supported their loved ones through adult mental health services, meaning they could not provide experiences of CEDS-CYP. Future research should seek to explore if there are disparities between CEDS-CYP and adult services, as well as between out-patient, day care and in-patient settings, in adhering to the Beat and AED publications.

Despite data saturation being reached, our sample was small and lacked diversity. All of our sample self-reported ethnicity as White, and most loved ones with an eating disorder were female, aged 21–30 years and had anorexia nervosa, making it hard to ascertain if our findings are generalisable to wider sibling and partner caregiving groups. To increase the diversity of our sample, we aim to widen access to this research project via our national online survey. We aim to recruit individuals from diverse cultural and socioeconomic backgrounds, and well as those caring for loved ones in underrepresented eating disorder groups, such as men and older women.^20^

Given the online recruitment and focus group facilitation methods utilised, participation may not have been possible for carers without access to appropriate devices, poor internet connectivity or a lack of a private space. Additionally, as participants were provided with a £20 voucher for participation, some participants may have presented as carers for monetary gain, resulting in potential participant misrepresentation and potential reduction in sample validity. To address this risk, recruitment through eating disorder services may be beneficial in future research. Moreover, our findings may be biased as our participants may have reflected those with negative service experiences who were more motivated for change.

Overall, this qualitative focus group study, which captured the experiential perspectives of siblings and partners of a loved one with an eating disorder, identified that the best-practice standards and healthcare rights published by Beat and AED are not being fulfilled. Notably, several changes for improving support for siblings and partners were identified, including a proactive approach from services to detect difficulties, promote skills training, provide emotional/practical support and tailored peer support groups, and support online facilitation, which could be delivered either in-house or through signposting to the charity sector, such as Beat. These findings have been used to inform the development of a large-scale national online survey to identify the experiences and needs of carers of individuals with eating disorders.

## Data Availability

The data that support the findings of this study are available from the corresponding author, H.C., upon reasonable request. The data are not publicly available due to containing information that could compromise the privacy of research participants.

## Appendix 1 Focus Group Topic Guide

Introduction and overview

- Welcome and introductions to research team
- Overview of the aims of the study:
  - ‘Our overall aim is to develop a national survey to improve understanding of the experience of close others of patients with eating disorders. From doing so, we hope to identify areas of improvement. Today, we will be using your experiences to help us to develop this survey. We will be using 2 sets of recommendations outlined by Beat, the UK charity of eating disorders, and the Academy for Eating Disorders. We would like to know whether your lived experiences align with these guidelines. This will inform the development of our national survey.’
- Instructions regarding the focus group:
  - ‘There are no right or wrong answers – all your responses are valid. Please give everyone a balanced use of airtime. Please help to protect others’ privacy by not discussing details outside of today's focus group. Please don't use identifiable information in the discussions (e.g., your loved one's name). We are recording the discussion – once you have said something, you cannot erase it from the recording so please only share what you are comfortable with. Please try to stay on topic – we only have one hour!’

Materials

- All participants are provided a pack of materials, which includes:
  - Consent form
  - Information sheet
  - Participant demographics form
  - A copy of the Beat recommendations
  - A copy of the Academy for Eating Disorder recommendations

Discussion points (approximately 10 min per discussion point)

1. Which recommendations are you aware of or familiar with?
2. Focusing on the recommendations from Beat, please choose the top 3 most important recommendations
3. Looking at the top 3 recommendations:
  - What would you consider good support for carers of people with eating disorders?
  - How do they match your personal experience?
4. What other good practice for carers have you experienced?
5. What do you want out of the national survey?
